# A hybrid absorption–adsorption method to efficiently capture carbon

**DOI:** 10.1038/ncomms6147

**Published:** 2014-10-09

**Authors:** Huang Liu, Bei Liu, Li-Chiang Lin, Guangjin Chen, Yuqing Wu, Jin Wang, Xueteng Gao, Yining Lv, Yong Pan, Xiaoxin Zhang, Xianren Zhang, Lanying Yang, Changyu Sun, Berend Smit, Wenchuan Wang

**Affiliations:** 1 State Key Laboratory of Heavy Oil Processing, China University of Petroleum, Beijing 102249, P. R. China; 2 Department of Chemical and Biomolecular Engineering, University of California, Berkeley, California 94720, USA; 3 State Key Laboratory of Organic-Inorganic Composites, Beijing University of Chemical Technology, Beijing 100029, P. R. China; 4 Laboratory of Molecular Simulation, Institut des Sciences et Ingénierie Chimiques, Ecole Polytechnique Fédérale de Lausanne (EPFL), CH-1015 Lausanne, Switzerland

## Abstract

Removal of carbon dioxide is an essential step in many energy-related processes. Here we report a novel slurry concept that combines specific advantages of metal-organic frameworks, ion liquids, amines and membranes by suspending zeolitic imidazolate framework-8 in glycol-2-methylimidazole solution. We show that this approach may give a more efficient technology to capture carbon dioxide compared to conventional technologies. The carbon dioxide sorption capacity of our slurry reaches 1.25 mol l^−1^ at 1 bar and the selectivity of carbon dioxide/hydrogen, carbon dioxide/nitrogen and carbon dioxide/methane achieves 951, 394 and 144, respectively. We demonstrate that the slurry can efficiently remove carbon dioxide from gas mixtures at normal pressure/temperature through breakthrough experiments. Most importantly, the sorption enthalpy is only −29 kJ mol^−1^, indicating that significantly less energy is required for sorbent regeneration. In addition, from a technological point of view, unlike solid adsorbents slurries can flow and be pumped. This allows us to use a continuous separation process with heat integration.

## Abstract (web summary)

Common approaches for carbon dioxide capture include absorption in amine solutions or adsorption in porous materials. Here, the authors combine these two strategies and report carbon dioxide capture in a slurry of metal-organic frameworks in glycol-2-methylimidazole, which has advantages of both techniques.

## Graphical abstract

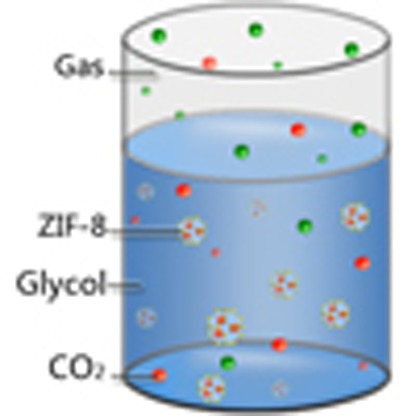

Reduction of CO_2_ emission is directly influenced by the efficiency with which we are able to capture carbon from flue gas and other gas mixtures related to energy generation, such as biogas, integrated gasification combined cycle gas, syngas, shift gas produced from steam reforming of natural gas or coal and natural gas^1^,^2^,^3^,^4^. Motivated by the staggering energy costs associated with these gas separations, finding the optimal material for a given separation has become a very active area of modern chemistry^5^,^6^,^7^. In particular, metal-organic frameworks (MOFs)^8^, a new class of crystalline nanoporous materials, are regarded as promising candidates for CO_2_ separations. MOFs are three-dimensional networks of metal clusters that are connected with organic linkers; by changing the metal and/or linker we can synthesize millions of different materials. Moreover, one can use this tunability to synthesize a material that has exactly the right pore volume, surface area and selectivity to efficiently separate CO_2_ (refs 9, 10, 11).The enthusiasm of the scientific community about MOFs and other nanoporous materials as solid adsorbents, however, does not yet resonate in the process engineering community^12^. To understand why not, consider a simple solid-adsorption separation process for carbon capture from flue gas. The first step involves an adsorber, containing the nanoporous material, which selectively adsorbs CO_2_ from the flue gas. Once the adsorber is saturated, regeneration is required, which is typically done by supplying heat (temperature swing adsorption) or applying vacuum (pressure swing adsorption) to the adsorbent. This process needs at least two columns, which alternate between the adsorption and the regeneration mode. Liquid absorption uses a similar process, replacing the nanoporous material with, for example, an amine solution. If we now compare one of the most promising MOFs with commercially available amine solutions, the energy required to regenerate the amine solutions is about one order of magnitude larger than the energy required to regenerate this MOF^4^,^13^. This is because in amine solutions, the CO_2_ is so strongly bound that one needs to boil the amine solution to reverse the chemical bonding; as the amine solution contains 70% water, most of this energy is actually used for boiling water. Nevertheless, liquid absorption is the current state-of-the-art process for carbon capture and, surprisingly, solid adsorption is not considered as such a promising alternative. The fact is due to two significant advantages of the use of liquids. First, the liquid phase allows us to use advanced heat integrations to recover a large fraction of the heat. In solid adsorption by contrast, efficient heat integration is very difficult. Without heat recovery, the energy efficiency of a solid-adsorption process will be low. Second, pumping allows liquid absorption to be carried out in a continuous process, whereas the solid adsorbent is typically used in a less efficient batch process. To take full advantage of the exciting developments in the field of MOFs, it is essential to remove these intrinsic difficulties related to a solid-adsorbent process.

The approach we develop in this work is based on the idea that we can make slurries by suspending solid adsorbents in a liquid absorbent. From a process engineering point of view, slurries are very similar to liquids. Hence, with slurries we can develop a continuous process and use heat integration. For conventional adsorbents, slurries would be a terrible idea, as the liquid would fill the pores. The beauty of MOFs is, however, that we can use their tunability to select a material with pores that are sufficiently small to prevent liquid-absorbent molecules from entering the materials, but sufficiently large for the gas molecules to be adsorbed. A carefully chosen absorbent/adsorbent combination may lead to a significantly enhanced separation performance, which is denoted as the absorption–adsorption system.

## Results

### Separation with ZIF-8/glycol slurry

**Figure 1:**
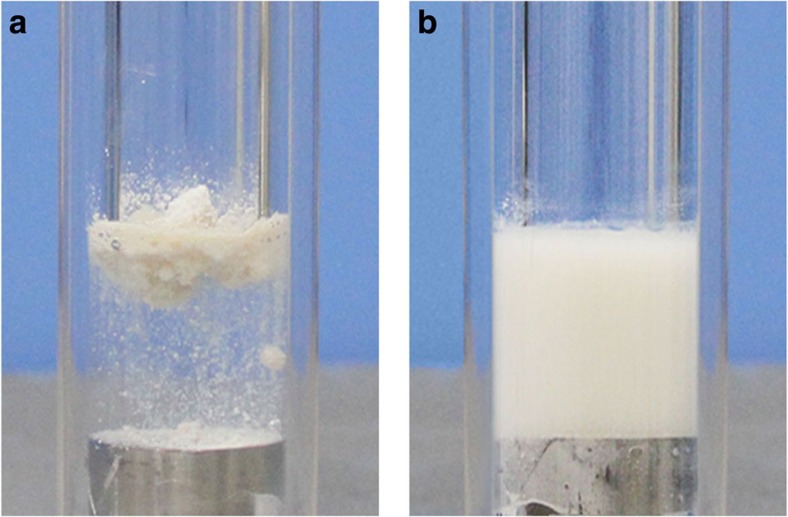
Formation of ZIF-8/glycol slurries. Photographs of experimentally prepared slurries with solid ZIF-8 suspended in liquid glycol: (**a**) before mixing, (**b**) after mixing.

**Figure 2:**
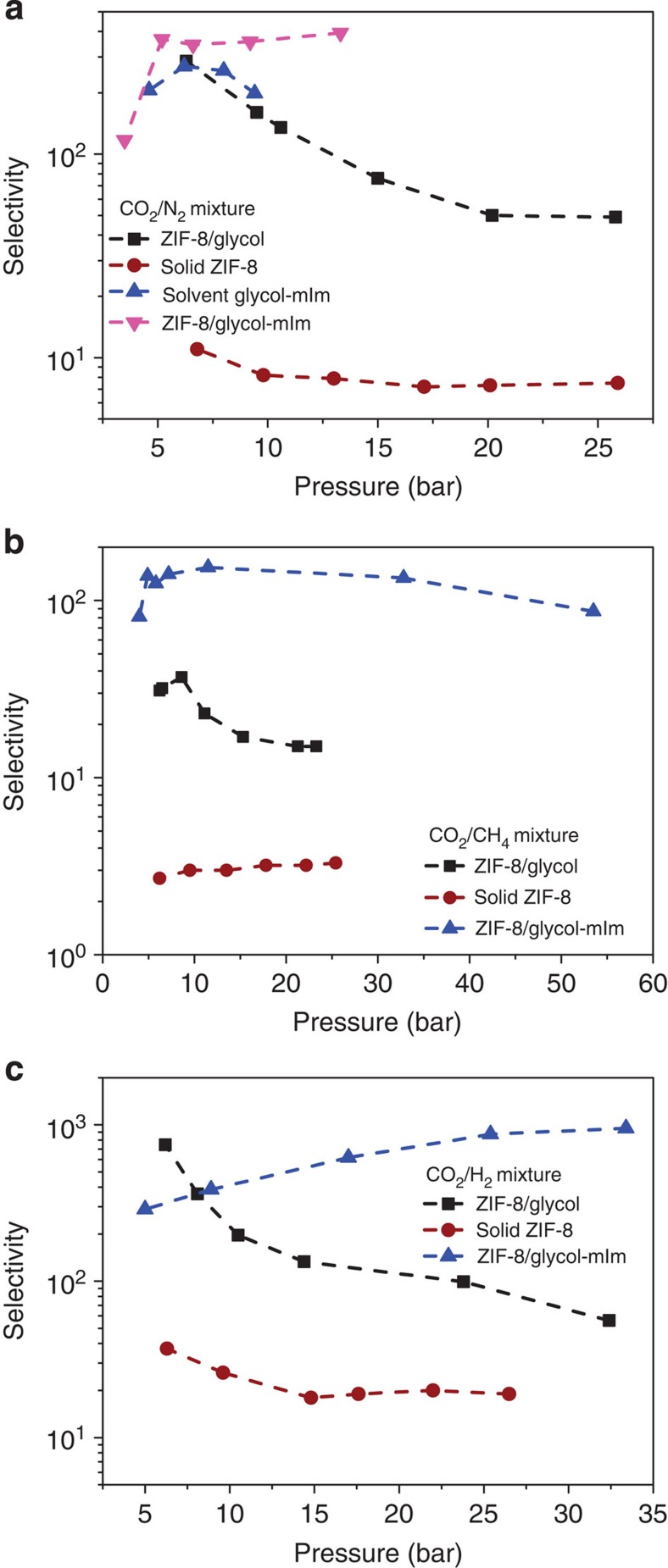
Separation performance of different media. This figure shows the selectivity of CO_2_ over (**a**) N_2_, (**b**) CH_4_ and (**c**) H_2_ as a function of pressure in the solid ZIF-8, ZIF-8/glycol slurry at 293.15 K and in liquid glycol–mIm, ZIF-8/glycol–mIm slurry at 303.15 K.

As a proof of concept, we first studied slurries of ZIF-8 (zeolitic imidazolate framework-8) suspended in liquid glycol (see Fig. 1). ZIFs are a sub-class of MOFs^14^, and generally possess excellent solution, chemical and thermal stability^15^,^16^,^17^. ZIF-8 has narrow six-membered ring pore windows (3.4 Å), which are smaller than the kinetic diameter of glycol molecules (4.5 Å)^18^, and hence glycol is unlikely to influence the adsorption capacity of CO_2_ in suspended ZIF-8. To demonstrate that our ZIF-8/glycol slurry can separate different CO_2_ mixtures, we performed adsorption measurements on different CO_2_ gas mixtures (that is, CO_2_ with CH_4_, N_2_ or H_2_, see Supplementary Tables 1–5). Figure 2 shows the selectivity as a function of pressure for these three mixtures (that is, up to ~745 for CO_2_/H_2_, ~286 for CO_2_/N_2_ and ~37 for CO_2_/CH_4_), which are sufficiently large for an effective separation. No remarkable loss of separation ability was observed after several times of cycling use of the slurry (Supplementary Table 2), where the slurry was regenerated by applying vacuum. Further characterization of the solid ZIF-8, recovered from the slurry after these cycles showed that the ZIF-8 structure remained intact (see scanning electron microscopy images, X-ray diffraction (XRD) patterns, Fourier transform infrared spectra, Fourier transform Raman spectra and energy dispersive X-ray spectroscopy patterns in Supplementary Figs 1–5). In addition to glycol, other liquids such as ethanol, cyclohexane, normal hexane, methylbenzene, tetrachloromethane and triethylene glycol have been tested to form slurries with ZIF-8 to separate a CO_2_/N_2_ gas mixture (Supplementary Table 6). It was found that only triethylene glycol is effective with a selectivity higher than 50. Compared with triethylene glycol, the other studied liquids consist of smaller molecules that can enter into ZIF-8 frameworks. Note that, to provide a reference for showing the superiority of the slurry approach proposed in this work, absorption separation using pure glycol or water for the CO_2_/N_2_ and CO_2_/CH_4_ gas mixtures (Supplementary Table 7) and adsorption separation using solid ZIF-8 for the CO_2_/N_2_, CO_2_/H_2_ and CO_2_/CH_4_ gas mixtures (Fig. 2, Supplementary Tables 8–10) were also performed.

### Separation with ZIF-8/glycol-2-methylimidazole slurry

**Figure 3:**
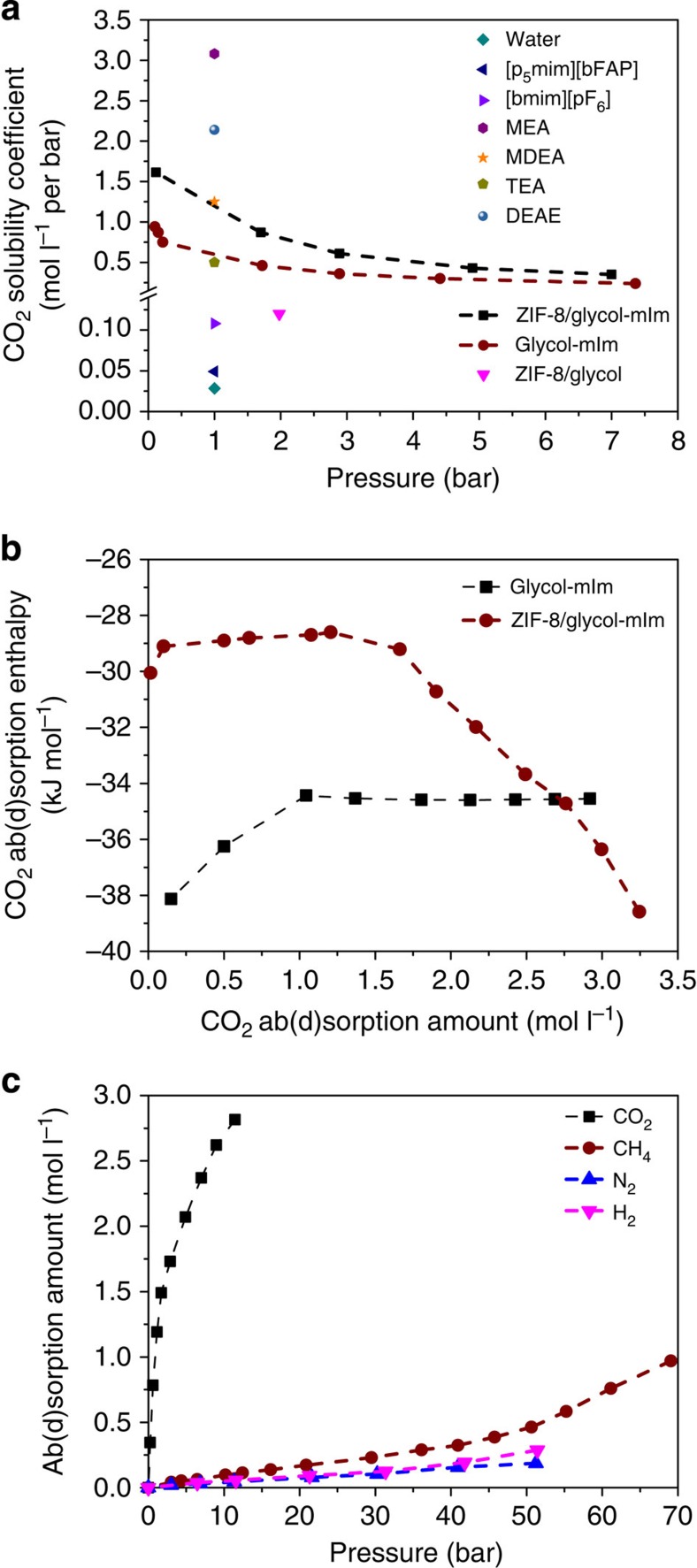
Ab(d)sorption solubility and enthalpies. (**a**) The comparison of CO_2_ solubility coefficients in ZIF-8/glycol–mIm slurry at 303.15 K with that in ZIF-8/glycol at 293.15 K and that reported in the literature ((MEA (mass fraction 30%), MDEA (mass fraction 30%), TEA (mass fraction 30%), DEAE (mass fraction 30%)) at 313.15 K, ([p_5_mim][bFAP], [bmim][PF_6_]) at 298.15 K)^4^,^20^,^21^,^22^,^23^, (**b**) sorption enthalpies of CO_2_ in glycol–mIm liquid and ZIF-8/glycol–mIm slurry at 303.15 K (**c**) isotherms of CO_2_, CH_4_, N_2_ and H_2_ at 303.15 K in ZIF-8/glycol–mIm slurry.

Though we have obtained promising CO_2_ selectivities, Fig. 3a indicates that, as in most application the partial CO_2_ pressure is low, the solubility coefficient of ZIF-8/glycol slurry at these conditions is too low for practical applications. We further tuned the absorbent by adding 2-methylimidazole (mIm) to the glycol. The solubility of CO_2_ in glycol–mIm (3:2) solution is 0.64 mol l^−1^ at 303.15 K and 1 bar (see Fig. 3a) and the selectivity of CO_2_ over N_2_ is higher than 200 (Supplementary Table 11). The CO_2_ absorption enthalpy in glycol–mIm solution is only about −34 kJ mol^−1^ at 303.15 K (Fig. 3b), which can lead to a much lower regeneration cost compared with many of the aqueous alkanolamines (around −100 kJ mol^−1^)^12^ and ion liquids^19^.

For the ZIF-8/glycol–mIm slurry system, we now obtain a solubility coefficient, *S*_c_, in (ZIF-8 15 wt%+mIm 34 wt%+glycol 51 wt%) of 1.63 mol l^−1^ per bar (Fig. 3a, Supplementary Fig. 6 and Supplementary Table 12). Such solubility is sufficiently high at low CO_2_ partial pressures to ensure a good sorption capacity at these conditions of practical interest. Although this solubility is still slightly smaller than that in MEA^20^ and MDEA^21^ solutions that are used in the current technologies, it is much higher than some promising ion liquids for CO_2_ capture: about 33 times higher than that in ion liquid [p_5_mim][bFAP] (0.048 mol l^−1^ per bar at 298.15 K)^4^ and about 15 times higher than that in ion liquid [bmim][PF_6_] (~0.108 mol l^−1^ per bar at 298.15 K)^22^. We anticipate that the solubility might further increase with higher ZIF-8 fraction (indicated by Supplementary Table 13). Furthermore, the CO_2_ absorption–adsorption enthalpy in the slurry is only about −29 kJ mol^−1^ (see Fig. 3b, which was obtained from Supplementary Fig. 7). Compared with the pure glycol–mIm liquid, the prepared slurry has an enhanced CO_2_ solubility with a reduced energy requirement for CO_2_ desorption. One can expect that an efficient CO_2_ capture process at lower energy cost can be achieved by using this advanced slurry in this regard.

Another important result is that the selectivity of the ZIF-8/glycol–mIm slurry is significantly higher than the selectivities observed in both ZIF-8/glycol slurry and glycol–mIm solution (see Fig. 2, Supplementary Tables 12–15). The highest selectivity for CO_2_/H_2_, CO_2_/N_2_ and CO_2_/CH_4_ reaches 951, 394 and 144, respectively, which are sufficiently large for an effective separation. To the best of our knowledge, these selectivities are better than those reported in MOFs^23^,^24^. Moreover, as shown in Fig. 2, such a high selectivity was obtained for a large pressure range, which demonstrates that this kind of slurry is suitable for the CO_2_ capture from different kinds of feed gases such as flue gas (CO_2_/N_2_, ~1 bar), biogas (CO_2_/CH_4_, ~1 bar), integrated gasification combined cycle gas (CO_2_/H_2_, 3~5 MPa) or natural gas (CO_2_/CH_4_, >5 MPa). To further quantify the performance of the ZIF-8/glycol–mIm slurry in a separation, we measured the absorption–adsorption isotherms for single gas components (CO_2_, CH_4_, N_2_ and H_2_). As seen from Fig. 3c, the uptake of CO_2_ in the slurry is much higher than those of other components, especially in lower pressure range. It should be noted that all measurements for both solubility and selectivity in the ZIF-8/glycol–mIm slurry shown in Figs 2 and 3 were performed using the same slurry through cycles of sorption/desorption (that is, apply vacuum for desorption). We observed no remarkable loss of the mass and separation ability of the slurry during these measurements. Similar to the ZIF-8/glycol system, further characterization of the solid ZIF-8, which were recovered from the slurry that has been reused by 33 times in 25 days, showed that the ZIF-8 structure remained intact (Supplementary Figs 1e and 2c). Supplementary Movie 1 illustrates the CO_2_ capture process (fresh slurry, equilibrium slurry, gas desorption process and recovered slurry).

### Column breakthrough tests

**Figure 4:**
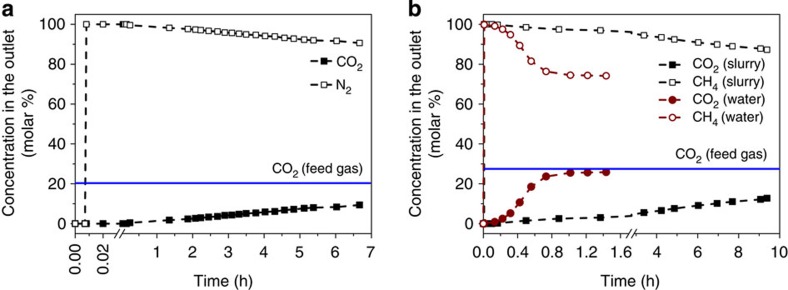
Breakthrough experiment. (**a**) Bubble column breakthrough experiment for a CO_2_/N_2_ gas mixture (*z*_1_=0.2065) (303.15 K, 1 bar) carried out in ZIF-8/glycol–mIm slurry, (**b**) Column breakthrough experiments for a CO_2_/CH_4_ gas mixture (*z*_1_=0.276) (303.15 K, 1 bar) carried out in both ZIF-8/glycol–mIm slurry and pure water. The *x* axis is the time of the breakthrough experiment, the left *y* axis represents the concentration of gas components in outlet gas.

To mimic an actual separation process for capturing carbon in ZIF-8/glycol–mIm slurry, column breakthrough tests using two binary mixtures, CO_2_/N_2_ (*z*_CO2_=0.2065) and CO_2_/CH_4_ (*z*_CO2_=0.276), at 303.15 K were performed in a stainless bubbling column (Supplementary Fig. 8). For the CO_2_/N_2_ (*z*_CO2_=0.2065) mixture, N_2_ breakthrough occurred within 1 min, whereas CO_2_ breakthrough occurred after about 17 min for CO_2_/N_2_ (Fig. 4a and Supplementary Table 16). After the breakthrough of CO_2_, notably, the concentration of CO_2_ in the outlet gas still kept low for a long time; even after 6.7 h, the concentration of CO_2_ in the outlet gas was still less than half of the CO_2_ concentration in the feed gas. Similar results were obtained for CO_2_/CH_4_ (Fig. 4b and Supplementary Table 17). It should be noted that the slurry used for CO_2_/N_2_ was regenerated by purging with Helium at 318.15 K and atmospheric pressure, and then used for CO_2_/CH_4_. As the height of slurry in the column is only 1.31 m, the contact time of gas bubbles with slurry is very short. That suggests that this simple bubbling operation gives a high ab(d)sorption rate as most of CO_2_ in the inlet gas can be removed despite the short contacting time.

At this point, it is instructive to compare our ZIF-8/glycol–mIm slurry approach with the water-based technology that is currently used to separate CO_2_ from biogas. Biogas is a gas mixture of CO_2_ and CH_4_ coming from the breakdown of organic matter in the absence of oxygen^25^. To illustrate the superior performance of the absorption–adsorption approach compared with water, CO_2_/CH_4_ (*z*_CO2_=0.276) mixture breakthrough experiment using the slurry was compared with the conventional process based on water. Figure 4b (and Supplementary Table 18) shows that CO_2_ breakthrough occurred in the water-based system within 1 min, much faster than that in ZIF-8/glycol–mIm slurry (10 min). In addition, after about 1 h, the concentration of CO_2_ in the outlet gas nearly equalled to that in the inlet gas (27.6 mol%) for the water-based system, while for our slurry the CO_2_ concentration was only 3.04 mol%, and it increased to only 12.73 mol% after 9.4 h.

## Discussion

**Figure 5:**
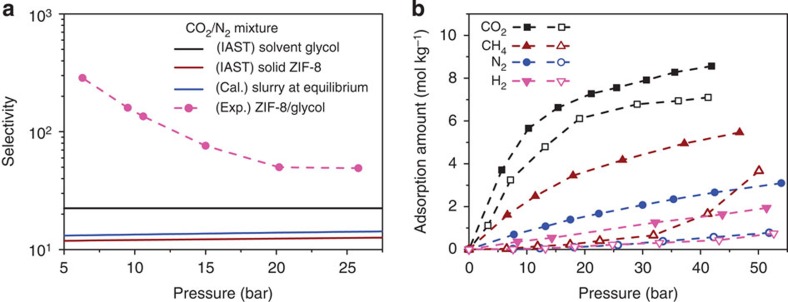
Enhanced selectivity in ZIF-8/glycol slurries. (**a**) The experimentally measured CO_2_/N_2_ selectivity at 293.15 K in ZIF-8/glycol slurries (solid circles). For comparison, we have calculated the expected selectivities in the pure solid ZIF-8 phase (black lines), the pure liquid glycol phase (red lines) and the corresponding weighted average (blue lines). In these calculations, we used the ideal adsorbed solution theory (IAST)^26^ to predict the mixture adsorption isotherm from the experimental single-component adsorption isotherms. For these calculations, we used 15% CO_2_ concentration in gas phase and a mass fraction of ZIF-8 equals to 0.152 in liquid glycol for the weighted average, (**b**) adsorption isotherms of CO_2_, CH_4_, N_2_ and H_2_ at 293.15 K in pure solid ZIF-8 (closed symbols) and the ZIF-8 suspended in ZIF-8/glycol slurry (open symbols).

A better understanding of the slurry system can play an important role in further improving the proposed slurry approach. It is interesting to compare selectivities in the slurry with those in the pure solid. Let us first, take a purely thermodynamic look and assume that gases in the three phases are in equilibrium. At equilibrium, there is equal temperature and equal chemical potentials of each of the components in the gas, liquid and solid phases. If we further assume that the liquid absorbent and solid adsorbent in the slurry do not influence each other, the expected selectivity of a particular mixture will be the weighted average of the selectivities in the (pure) liquid absorbent or (pure) solid adsorbent^26^. Surprisingly, Fig. 5a shows that the selectivity of the slurry phase for all mixtures are significantly higher than the selectivity observed in the pure solid ZIF-8 phase, the pure liquid phase as well as the weighted average value of them.

**Figure 6:**
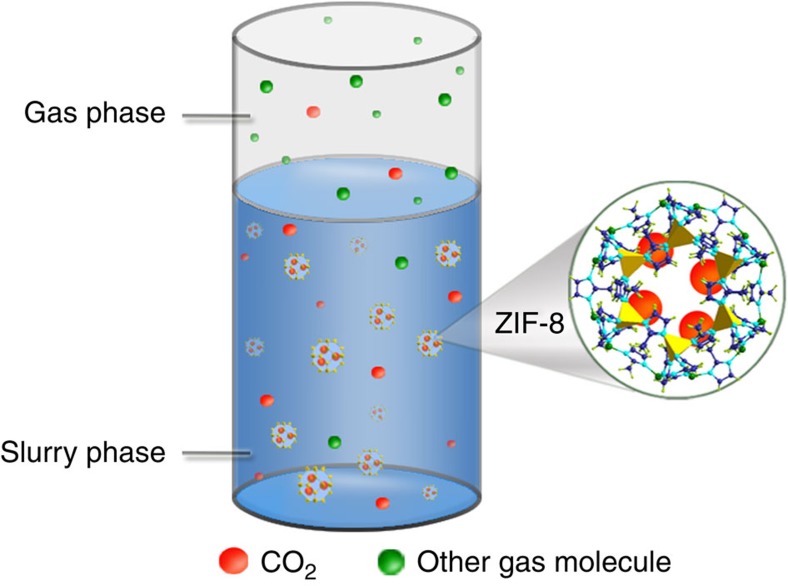
Hybrid absorption–adsorption process. Schematic of the hybrid absorption–adsorption separation process for CO_2_ gas mixtures in the slurry formed by ZIF-8 suspended in glycol solution.

To explain these observations, we have plotted in Fig. 5b the pure component isotherms inside the pure solid ZIF-8 and the ZIF-8 suspended in the slurry phase. These isotherms show that the adsorption of CO_2_ in the solid ZIF-8 suspended in a slurry is similar to adsorption observed in pure solid ZIF-8, but the corresponding adsorption of CH_4_, N_2_ and H_2_ is significantly lower. From the Henry constants of these gases in glycol at 293.15 K (Supplementary Table 19), we see that, compared with CO_2_, the solubility of the other gases is much lower. In the slurry, all gases that enter the solid phase need to first pass through the liquid film formed by glycol molecules surrounding solid particle. Therefore the low solubilities of CH_4_, N_2_ and H_2_ create significant mass-transfer limitations. The flux of these molecules is so low that on the relevant timescale the adsorption of these gases in ZIF-8 is far less than one can expect from equilibrium consideration and hence the selectivity in the slurry at low pressures is enhanced. Or stated differently, the liquid absorbent functions as a semipermeable membrane for which the permeability of those components other than CO_2_ is so low that, on the timescale of the separation, these components never reach the equilibration loading in the ZIF-8 suspended in the absorbent. A schematic diagram of the system can be seen in Fig. 6. If we increase the pressure (Fig. 2), we observe a decrease in the selectivity. These observations are consistent with an increase of the gas concentration in the liquid phase. On the other hand, if we decrease the temperature we see a further increase in selectivity (Supplementary Table 3). When we used glycol–mIm solution instead of pure glycol as solvent, we observed higher selectivity (Fig. 2a–c) as the solubility of CO_2_ in the glycol–mIm solution is much higher than that in pure glycol. At this point, it is instructive to point out that mIm is the single ligand used for synthesizing ZIF-8 and usually becomes the primary impurity of the product. Such an impurity has been shown to impose a seriously negative effect on the adsorption ability of solid ZIF-8 (ref. 27). However, as we have seen from this study, it has strong positive effect when ZIF-8 is used in slurry state. The lessened demand of product purity makes the production of ZIF-8 much less expensive, because the removal of surplus mIm by vapourizing the product requires high cost of energy.

To interpret the high CO_2_ absorbing ability of the glycol–mIm solution, we propose a mechanism as illustrated in Supplementary Fig. 9. We suggest that there is a quasi-chemisorptive formation of rather unstable N–C bonded carbamate species between CO_2_ and mIm. The rather unstable carbamate leads to a smaller CO_2_ absorption enthalpy in glycol–mIm solution, as it is usually much larger in typical chemical absorption^12^ or chemical adsorption^28^ processes. In aqueous amine, carbamate can further react with water and subsequently lead to quite stable carbonate species^29^. In the glycol–mIm solution, however, the carbamate species cannot further react with glycol. The mechanism is indirectly supported by experimental observations that the presence of CO_2_ can obviously increase the quantity of mIm dissolved in glycol. It should be noted that other researchers also proposed a similar mechanism for the absorption of CO_2_ in ion liquids that have function groups of −NH or −NH_2_ (refs 30, 31). In addition, there might be a structural interplay between glycol and mIm, which effectively lower the absorption enthalpy. A more detailed investigation, however, is required to validate the proposed mechanism and to better understand the fundamental physics/chemistry behind the system. In particular, as a future work, experimental characterizations, start-of-the-art quantum mechanical calculations and molecular simulations will be carried out to study the pure solvent and slurry systems in detail.

Three important insights emerge from this work. The unique properties of MOFs allow us to develop a slurry-based process to mitigate the main difficulties in the conventional solid-adsorption processes. As the slurry can be pumped, we can carry out a process very similar to liquid absorption. Second, the absorbent–adsorbent combination gives a surprisingly extra dimension for optimizing the design of a separation. The absorbent can act as a semipermeable membrane, preventing equilibration of one of the components and hence enhancing the selectivity of the separation. Finally, the single ligand mIm used in ZIF-8 synthesis (that is, also the main impurity of ZIF-8) could substantially increase the carbon capture capacity of the MOF/solvent-ligand slurry. It suggests a lower requirement on the MOF purities as well as a reduced cost in their production while using the slurry approach. In fact, in our example, we have chosen ZIF-8 that has a very modest selectivity towards CO_2_, yet in the slurry shows excellent separation performance because of this enhancement of the selectivity caused by the absorbent and mIm that is a general ligand in the synthesis of MOFs and ion liquids, yet in the slurry significantly increases the slurry’s carbon capture and separation efficiency. All of the above suggests that this work opens up many exciting possibilities to further optimize CO_2_ removal process by combining the specific advantages of MOFs, ion liquids, amines and membranes.

## Methods

### Materials

Materials used in this work include ZIF-8, mIm, glycol, ethanol, *n*-hexane, cyclohexane, methybenzene, tetrachloromethane, triethylene glycol, water and feed gases. Among them, both ZIF-8 and mIm were purchased from Sigma-Aldrich. The scanning electron microscopy images (Supplementary Fig. 1a) and XRD patterns of the purchased ZIF-8 sample (Supplementary Fig. 2a) are in good agreement with the ones reported in the literature^32^. Glycol, ethanol, *n*-hexane, cyclohexane, methylbenzene, tetrachloromethane and triethylene glycol were purchased from Beijing Chemical Reagents Company, China. Analytical grade carbon dioxide (99.99%), nitrogen (99.99%), methane (99.99%) and hydrogen (99.999%) were purchased from Beijing AP Beifen Gas Industry Company, China. The synthetic gases CO_2_/N_2_, CO_2_/H_2_ and CO_2_/CH_4_ were prepared in our own laboratory. A Hewlett-Packard gas chromatograph (HP 7890) was used to analyze the composition of the prepared gases.

### Ab(d)sorption measurements

All the ab(d)sorption measurement experiments were performed using the experimental apparatus as schematically illustrated in Supplementary Fig. 10. A detailed description of the setup can be found in our previous report^33^. The key parts of the apparatus are a transparent sapphire cell and a steel-made blind cell, which are both installed in an air bath. The effective volume of the sapphire cell is 60 cm^3^ and that of the blind cell plus connected tubes is 112 cm^3^. The maximum working pressures of these two cells are designed to be 20 MPa. To directly observe samples in the cell, a lamp with luminescence source (type LG100H) is mounted on the outside of the cell. A secondary platinum resistance thermometer (type-pt100) is used as the temperature sensor. A calibrated Heise pressure gauge and differential pressure transducers are used to measure the system pressure. The uncertainties of pressure and temperature measurements are ±0.01 MPa and±0.1 K, respectively. Real-time readings of the system temperature and pressure are recorded.

Before the experiments, the sapphire cell was dismounted from the apparatus, washed with distilled water and dried, then loaded with a known quantity of dry porous material. After that, a known amount of solvent was immersed into the sapphire cell slowly and evenly. Both the used dry porous material and solvent were weighed by an electrical balance with a precision of ±0.1 mg. The mixture of porous material and liquid solvent was stirred to form a suspension mixture (that is, slurry). Subsequently, the cell was installed back into the apparatus. The system (sapphire cell+blind cell+tubes connecting two cells) was then purged through vacuuming. Enough amount of synthetic gas was injected into the blind cell, then the desired value of temperature was set through the air bath. Once both temperature and pressure of the blind cell were kept constant, the pressure of gas mixture in the blind cell was recorded as the initial pressure *P*_0_. The top valve of the sapphire cell was opened slowly then, letting the desired amount of synthetic gas flow into the sapphire cell from the blind cell. Afterwards, this valve was closed and the magnetic stirrer was turned on. The pressure of the residual gas mixture in the blind cell was recorded as *P*_1_. With the sorption of gas mixture by the slurry, the system pressure in the sapphire cell decreased gradually. During each measurement, the pressure in the sapphire cell as a function time was recorded. When the system pressure remained as a constant for at least 2 h, we considered the equilibrium of system was achieved. The equilibrium pressure of the sapphire cell was recorded as *P*_E_. Gas mixture in the equilibrium gas phase of the sapphire cell was sampled under constant pressure by pushing the connected hand pump and analysed by a HP 7890 gas chromatograph. The volume of the slurry in the sapphire cell can be obtained by measuring the height of the equilibrium liquid phase. The inner radius of the sapphire cell is known to be 1.27 cm. In this work, the amount of each gas species absorbed and adsorbed in the measured sample was determined through mass balance as described below.

The total mole number of gas mixtures (*n*_t_) that was injected into the sapphire cell is calculated by the following formula:


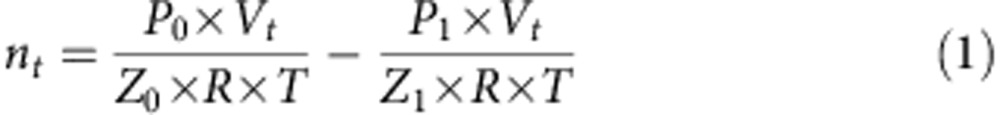


where *T* is the system temperature, *P*_0_ is the initial pressure of the blind cell, *P*_1_ is the equilibrium pressure of the blind cell after injecting gases into the sapphire cell, *V*_t_ is the total volume of the blind cell plus tubes connecting to it and *R* is the gas constant. Compressibility factors *Z*_0_ and *Z*_1_ were calculated using the Benedict–Webb–Rubin–Starling equation of state. The total gas amount (*n*_E_) in the equilibrium gas phase of the sapphire cell after absorption and adsorption equilibrium is determined by:


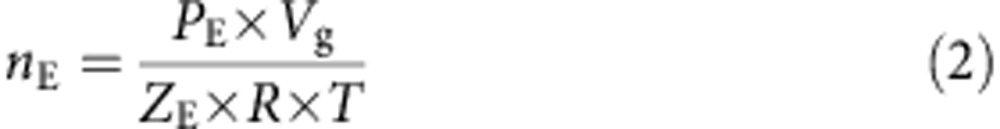


where *P*_E_ is the equilibrium pressure of the sapphire cell and *Z*_E_ is the compressibility factor corresponding to *T*, *P*_E_ and gas composition. *V*_g_ is the volume of equilibrium gas phase in the sapphire cell at the end of each experimental run. The total uptake of CO_2_ (*n*_1_) and that of N_2_ (CH_4_ or H_2_) (*n*_2_) in slurry are calculated as follows:


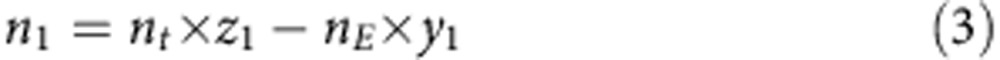



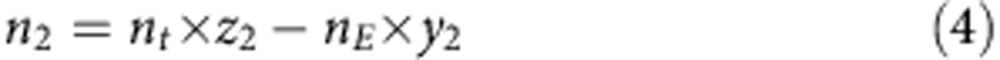


where *z*_1_ and *y*_1_ are the mole fraction of CO_2_ in the synthetic gas and equilibrium gas phase, respectively; *z*_2_ and *y*_2_ are the mole fraction of N_2_ (CH_4_ or H_2_) in the synthetic gas and equilibrium gas phase, respectively.

Accordingly, the apparent mole fractions of CO_2_ (*x*_1_) and N_2_ (CH_4_ or H_2_) (*x*_2_) in the equilibrium slurry phase can be obtained by the following formulas:


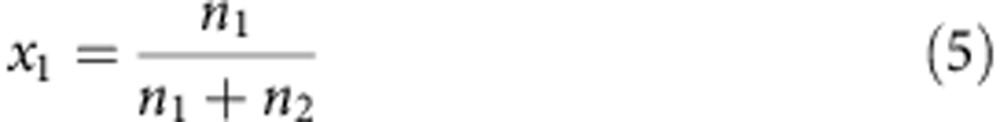



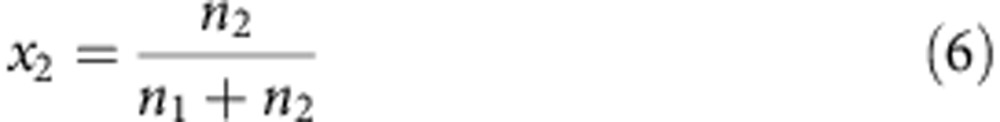


In an equilibrium-based separation process, a good indication of the CO_2_ separation efficiency is the sorption selectivity^34^. In this study, the apparent selectivity of CO_2_ over other component in slurry (*β*) is calculated as:


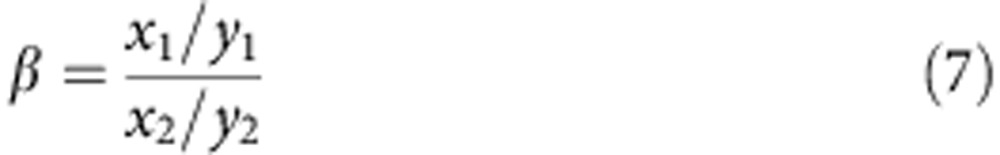


To provide a reference for showing the superiority of the proposed absorption–adsorption hybrid method, the selectivity of CO_2_ in solid ZIF-8 (*β*^′^) is also provided, which is calculated as:


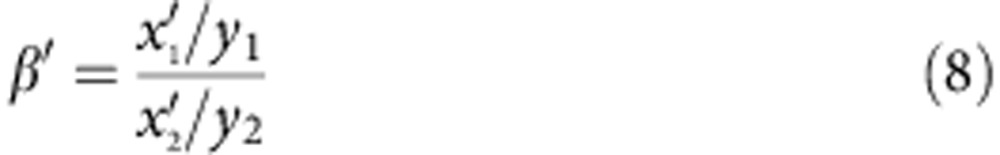


where 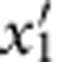 and 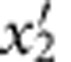 are the mole fractions of CO_2_ and N_2_ (CH_4_ or H_2_) in adsorbed phase (ZIF-8), respectively.

The solubility coefficient (*S*_c_) of CO_2_ in sorbents, an important indication of the separation capacity, is calculated as follows:


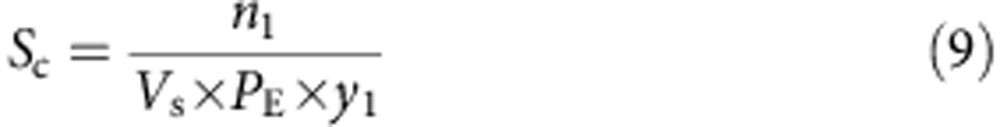


where *V*_s_ is the volume of sorbents (liquid or slurry).

The apparent volumetric solubility of CO_2_ in slurry (*S*_v_) is defined as:


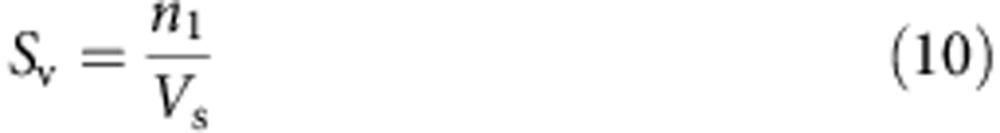


The initial gas–slurry volume ratio (*Φ*) and gas–solid adsorbent volume ratio (*Φ*′) are defined as:


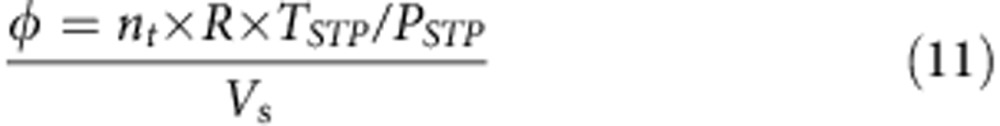



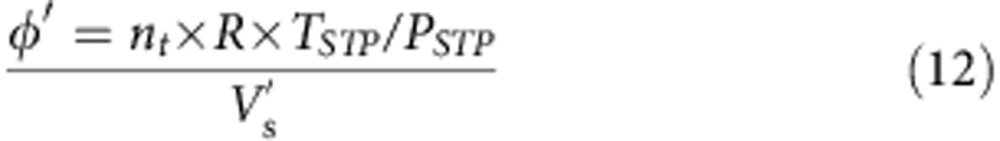


where 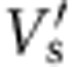 is the volume of solid adsorbent. *T_STP_* and *P_STP_* are standard temperature and pressure, respectively.

The adsorption capacity of solid ZIF-8 suspended in liquid absorbents for gas components is calculated as follows:


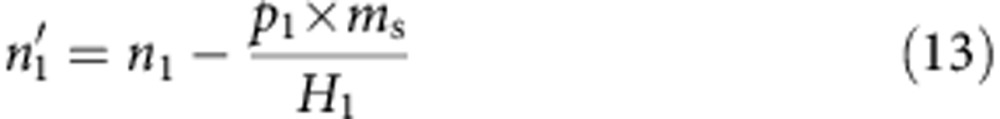



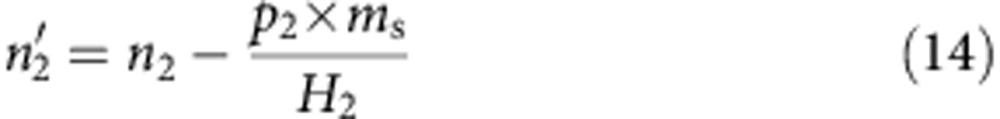


where 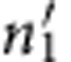 and 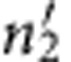 are the total uptake of CO_2_ and that of N_2_ (CH_4_ or H_2_) in ZIF-8 that is suspended in slurry, *H*_1_ and *H*_2_ are their Henry constants in liquid absorbents, and *p*_1_ and *p*_2_ are the corresponding equilibrium partial pressure, respectively. *m*_s_ is the mass of absorbents. Henry constants of CO_2_, CH_4_, N_2_ and H_2_ in glycol at 293.15 K are experimentally determined and reported in Supplementary Table 19.

The mass balance method stated above has been used in our previous work^33^,^35^. To validate our measurement approach, the adsorption isotherm of pure CO_2_ and the gas selectivity obtained for CO_2_/N_2_ (*z*_1_=0.2286) mixture in solid ZIF-8 at 303.15 K were measured using this method and compared with the literature data (Supplementary Figs 11 and 12).

### Characterization

The adsorbents (solid ZIF-8) were characterized by XRD (SIMADU XRD 6000) with Cu Kα radiation (0.1542, nm, 40 kV and 400 mA) at a scanning rate of 2 °C per minute. The morphologies and energy dispersive X-ray spectroscopy measurements were obtained using a FEI Quanta 200F scanning electron microscope. Fourier transform infrared spectra were obtained using a Bruker 80v spectrometer. Fourier transform Raman spectra were obtained using a HORIBA XploRA spectrometer. The thermogravimetric measurements were carried out at a NETZSCH STA 409 PC/PG instrument.

### Column breakthrough tests

The experimental setup used for dynamic breakthrough measurements is shown in Supplementary Fig. 8. The gas manifold consists of two lines, A and B, equipped with mass flow controllers. From line ‘A’, an inert gas (that is, helium) is injected to purge air out of the column and pipelines before experiment and to regenerate the slurry after each experiment. From line ‘B’, a gas mixture containing CO_2_ is injected with a constant flow rate for the breakthrough experiment. Both lines ‘A’ and ‘B’ are connected to the column using a three-way valve. The stainless column is 165 cm high with a jacket that is connected to a water batch for the temperature control. The inner diameter of the column is 2 cm. About 400 ml absorbent such as ZIF-8/glycol–mIm slurry is loaded in the column. A gas distributor was fixed at the bottom of the column. The designed maximum working pressure of this apparatus is 5 MPa. The outlet gas from the top of the column is sampled and analysed by a HP 7890 gas chromatograph.

## Author contributions

G.C., C.S. and B.S. designed the experiments; B.S. and G.C. led data analysis. H.L., Y.W., J.W., X.G., Y.L., Y.P., X.X.Z. and L.Y. performed laboratory experiments. B.L., L.-C.L., H.L., G.C., B.S., C.S., X.R.Z. and W.W. performed data analysis. H.L. and L.-C.L. prepared figures and tables. G.C., B.S., B.L., L.-C.L. and C.S. developed the project concept. L.-C.L., B.L., H.L., G.C., B.S., C.S. and W.W. wrote the manuscript.

## Additional information

**How to cite this article:** Liu, H. *et al.* A hybrid absorption–adsorption method to efficiently capture carbon. *Nat. Commun.* 5:5147 doi: 10.1038/ncomms6147 (2014).

## Supplementary Material

Supplementary Figures, Supplementary Tables and Supplementary References. Supplementary Figures 1-12, Supplementary Tables 1-19, Supplementary References.

Supplementary Movie 1 CO_2_ capture process in slurry
