# Supplementary material for: A hybrid absorption–adsorption method to efficiently capture carbon
Source: Nat Commun. 2014 Oct 9;5:5147. doi: 10.1038/ncomms6147 (PMC4214418; doi:10.1038/ncomms6147)
Supplement: Supplementary Figures, Supplementary Tables and Supplementary References. — Supplementary Figures 1-12, Supplementary Tables 1-19, Supplementary References. [file ncomms6147-s1.pdf]

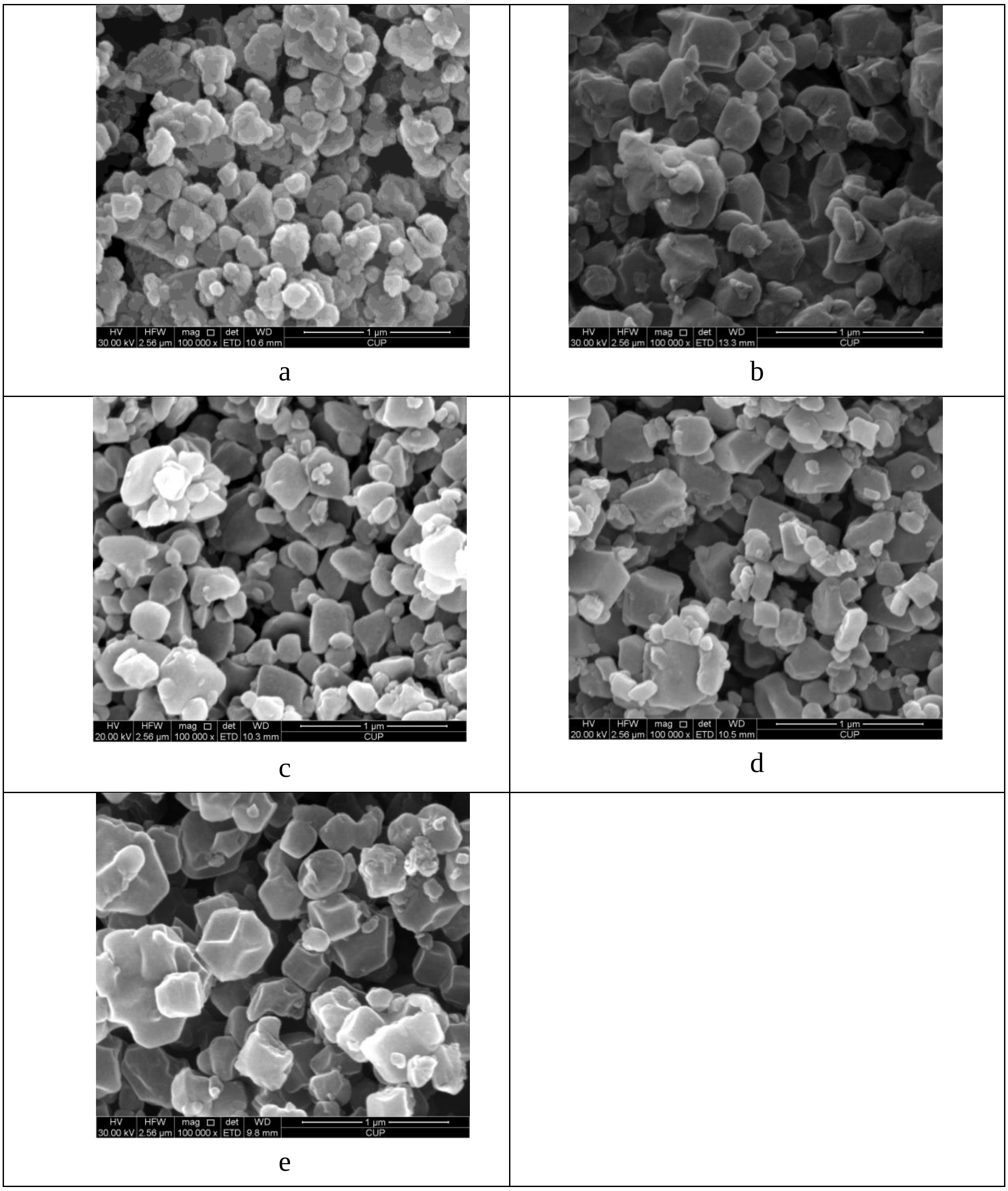

Supplementary Figure 1. Scanning electron microscopy (SEM) images Comparison of the SEM images of solid zeolitic imidazolate framework-8 (ZIF-8) between (a) fresh, recovered from ZIF-8/glycol slurry after being used for (b) one, (c) two, and (d) three times in separating CO_2/N_2 gas mixture (z_1=0.2231) at 293.15 K, (e) recovered from ZIF-8/glycol-mIm slurry that has been used for 33 cycles of sorption/desorption at 303.15 K within 25 days. The morphology of recovered ZIF-8 is as the same as that of fresh sample.

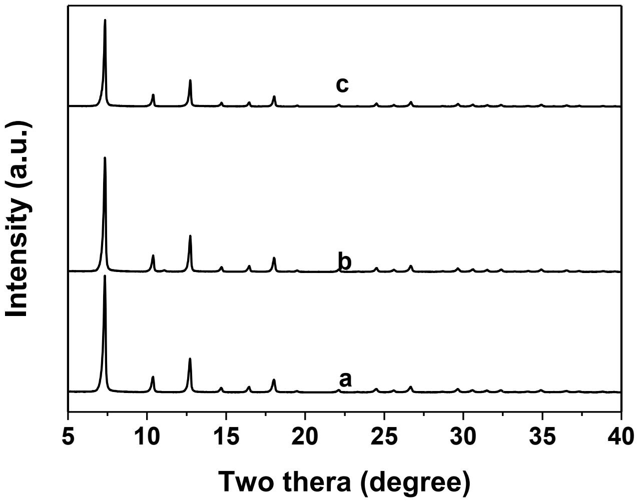

Supplementary Figure 2. Powder X-ray diffraction (PXRD) data Comparison of the experimental XRD patterns of solid ZIF-8 between (a) fresh ZIF-8, (b) recovered from ZIF-8/glycol slurry after being used for separating CO_2/N_2 gas mixture (z_1=0.2231), and (c) recovered from ZIF-8/glycol-mIm that has been used for 33 cycles of sorption/desorption at 303.15 K within 25 days. Y-axis of the graph shows the relative intensity. The comparison shows that the framework structure of recovered ZIF-8 is as the same as that of fresh sample.

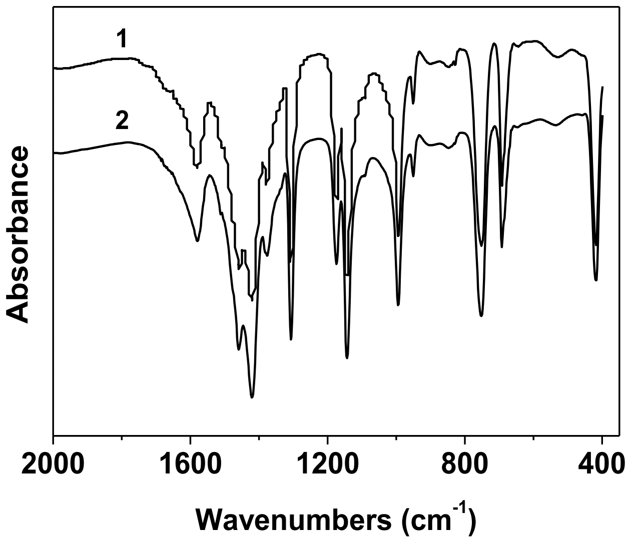

IR wavenumbers from 400 to 2000 cm^{-1}

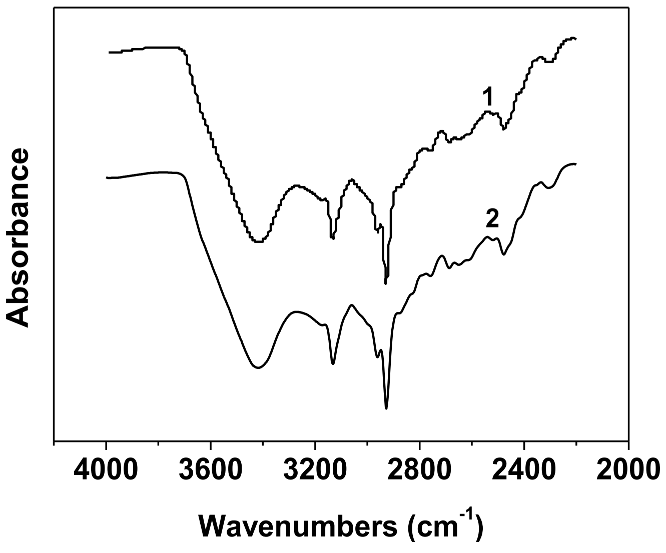

IR wavenumbers from 2000 to 4000 cm^{-1}

Supplementary Figure 3. Fourier transform Infrared (FT-IR) measurements Comparison of the FT-IR spectra of solid ZIF-8 between (1) fresh and (2) recovered from ZIF-8/glycol slurry after being used for separating CO_2/N_2 gas mixture (z_1=0.2231) at 293.15 K. The result shows that the framework structure of recovered ZIF-8 is as the same as that of fresh sample.

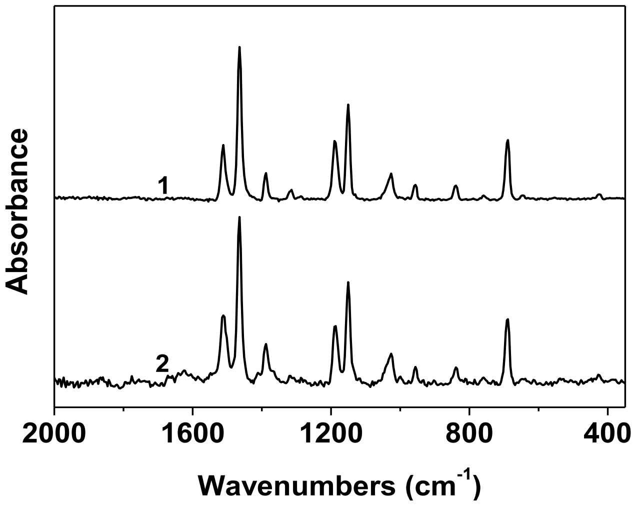

Raman wavenumbers from 400 to 2000 cm^{-1}

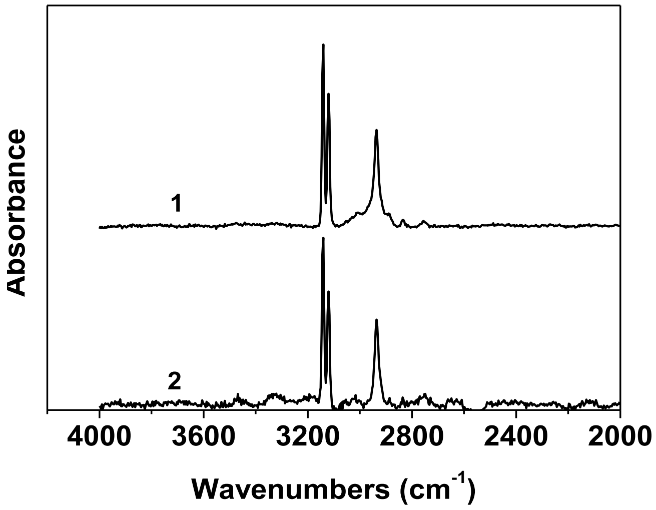

Raman wavenumbers from 2000 to 4000 cm^{-1}

Supplementary Figure 4. Fourier transform Raman (FT-Raman) measurements Comparison of the FT-Raman spectra of solid ZIF-8 between (1) fresh and (2) recovered from ZIF-8/glycol slurry after being used for separating CO_2/N_2 gas mixture (z_1=0.2231). The result shows that the framework structure of recovered ZIF-8 is as the same as that of fresh sample.

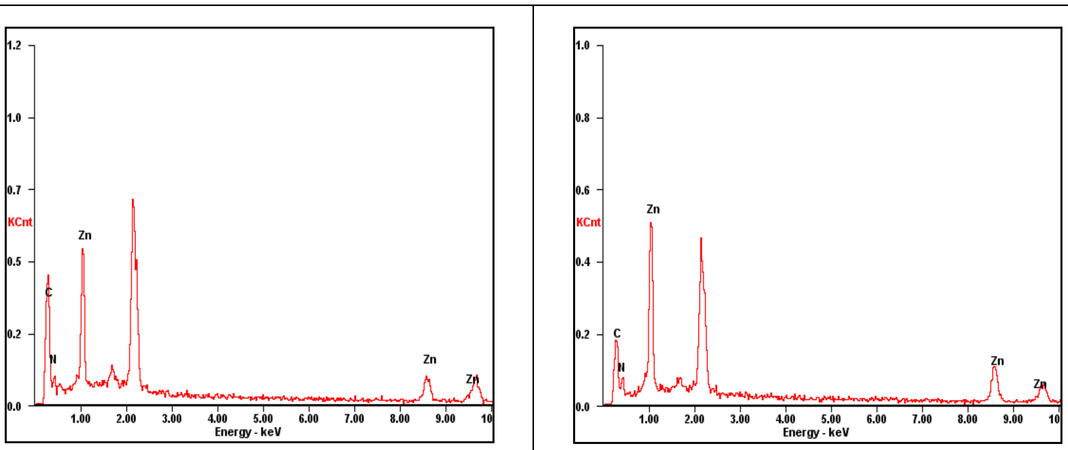

a b

Supplementary Figure 5. Energy dispersive X-ray (EDX) measurements EDX spectra of solid ZIF-8 between (a) fresh and (b) recovered from ZIF-8/glycol slurry after being used for one time. The elementary composition of recovered ZIF-8 from ZIF-8/glycol slurry is as the same as that of fresh sample.

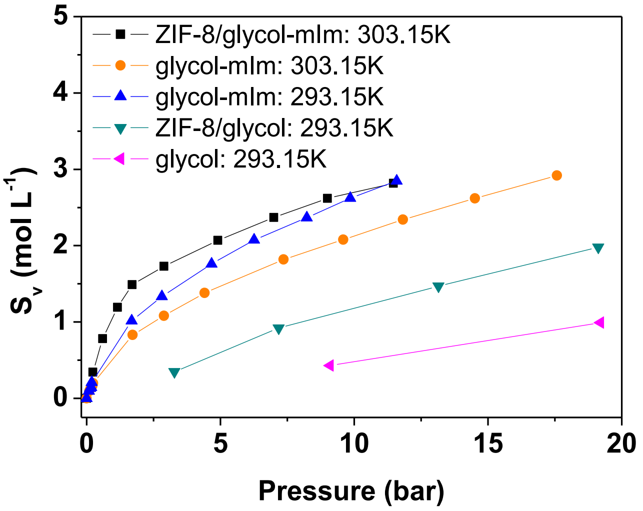

Supplementary Figure 6. CO_2 absorption isotherms The apparent volumetric solubility (S_v) of CO_2 in pure glycol, glycol-mIm (3:2) mixture, ZIF-8 (15.2 wt%)/glycol slurry, and ZIF-8 (15 wt%)/glycol-mIm (3:2) slurry at different temperatures. As can be seen, the order of S_v is ZIF-8/glycol-mIm > glycol-mIm > ZIF-8/glycol > glycol, demonstrating the superiority of absorption-adsorption hybrid method for CO_2 capture.

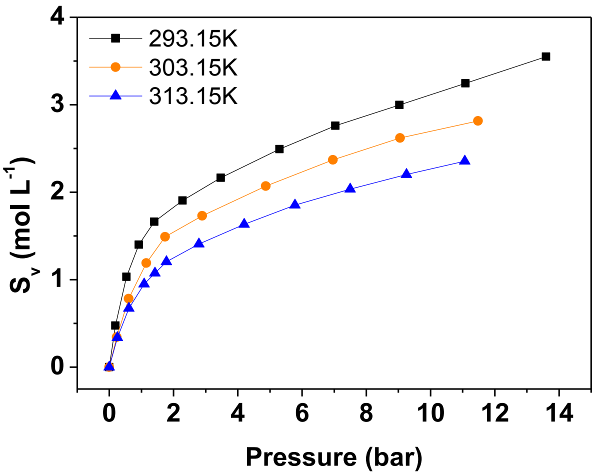

Supplementary Figure 7. Variable temperature isotherms The apparent volumetric solubility of CO_2 (S_v) in ZIF-8/glycol-mIm slurry at three different temperatures.

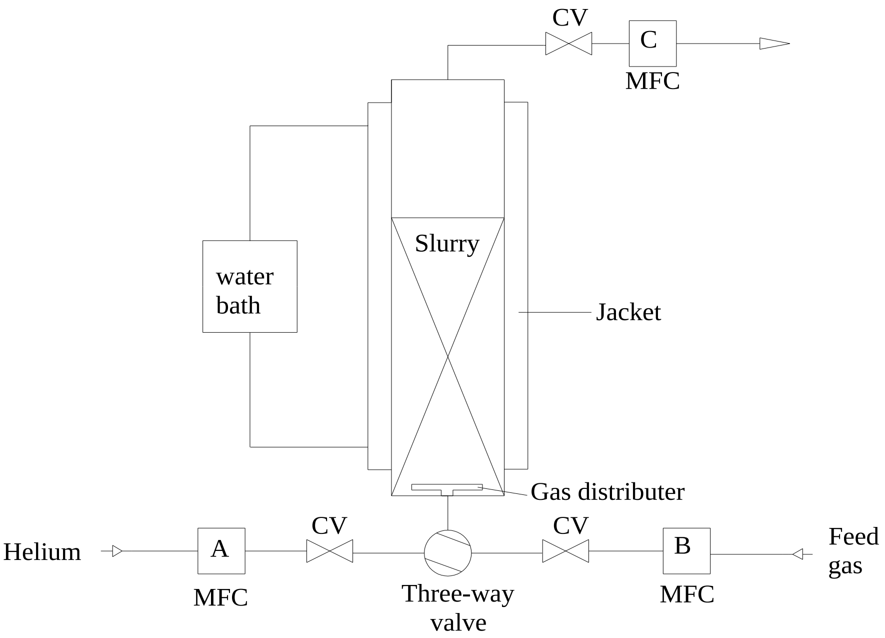

Supplementary Figure 8. Column breakthrough experiment. MFC stands for the mass flow controller.

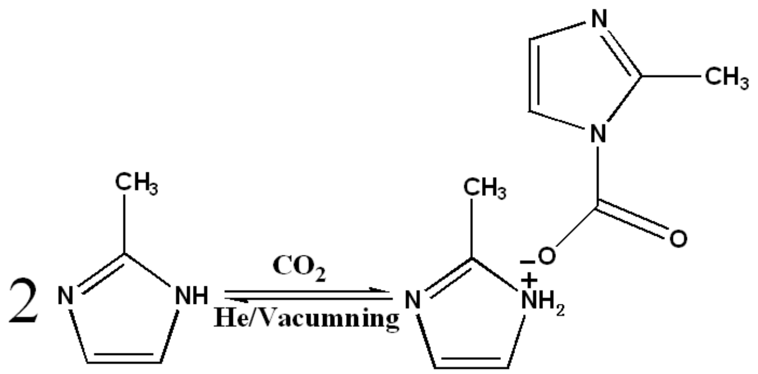

Supplementary Figure 9. Schematic for the absorption mechanism of CO_2 in glycol-mIm solution.

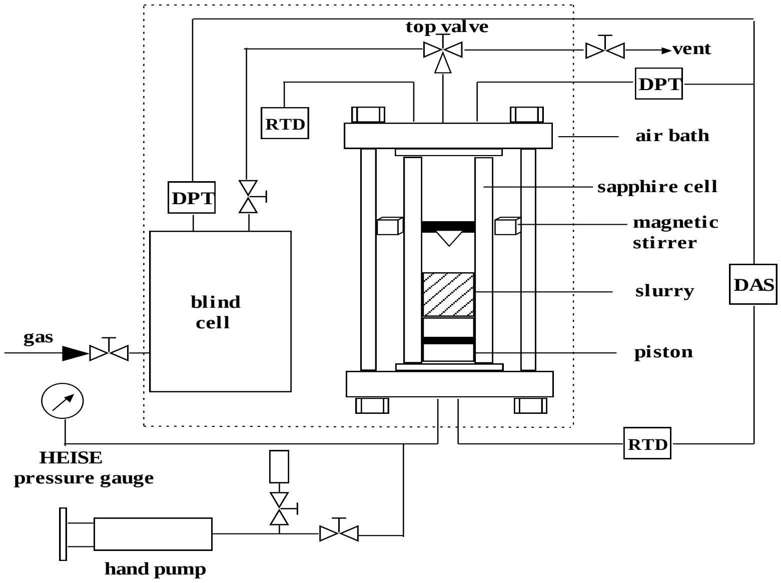

Supplementary Figure 10. Schematic diagram of the experimental apparatus. RTD, resistance thermocouple detector; DPT, differential pressure transducer; and DAS, data acquisition system.

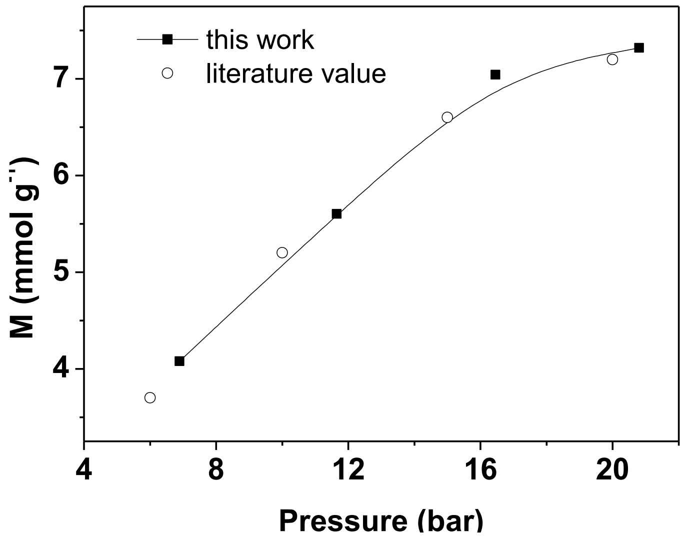

Supplementary Figure 11. CO_2 adsorption isotherms Comparison of the pure CO_2 adsorption isotherm in solid ZIF-8 at 303.15 K measured in this work and the one reported in literature[1].

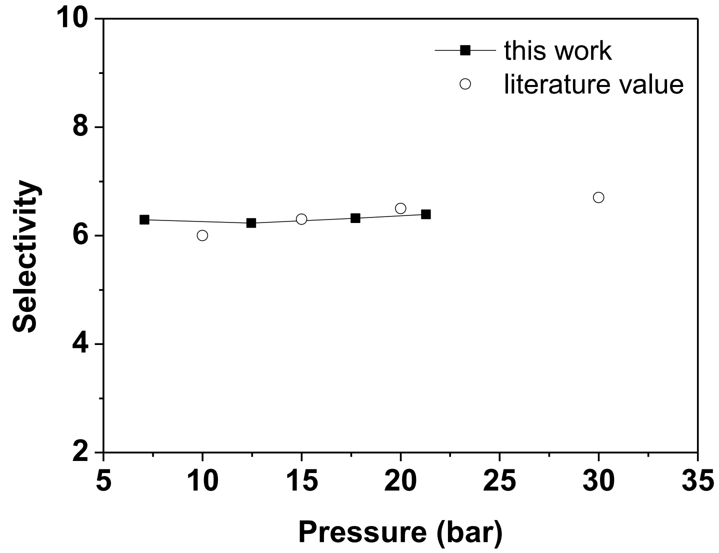

Supplementary Figure 12. CO_2/N_2 mixture on ZIF-8 Comparison of gas selectivities obtained for CO_2/N_2 (z_1=0.2286) mixture in solid ZIF-8 at 303.15 K measured in this work and the ones reported in literature[2].

Supplementary Table 1: CO_2/N_2 gas mixture (z_1=0.2231) separation results by using ZIF-8/glycol slurry with a ZIF-8 mass fraction of 0.152 at 293.15 K and different initial gas-slurry volume ratios Φ and initial pressures P_0.

P_0 (bar)	P_E (bar)	Φ	$P_{E\text{-}CO2}$ (bar)	y_1 (%)	x_1 (%)	β	S_c (mol L^{-1} bar^{-1})
7.14	6.25	18	0.58	9.22	96.68	286	0.21
12.41	10.56	31	1.05	9.89	93.69	135	0.18
17.58	15.00	45	1.52	10.10	89.54	76	0.18
23.25	20.17	59	2.34	11.58	86.81	50	0.14
28.71	25.79	75	3.06	11.85	86.87	49	0.13

Supplementary Table 2: CO_2/N_2 gas mixture (z_1=0.2231) separation results by using recycled ZIF-8/glycol slurries with a ZIF-8 mass fraction of 0.152 and nearly the same initial pressure of around 17.58 bar.

Reused times	P_0 (bar)	P_E (bar)	$P_{E\text{-}CO2}$ (bar)	y_1 (%)	x_1 (%)	β	S_c (mol L^{-1} bar^{-1})
0[a]	17.58	15.00	1.52	10.10	89.54	76	0.18
1	17.82	15.33	1.79	11.67	91.16	78	0.14
2	17.75	14.96	1.37	9.18	89.84	88	0.21
3	18.00	15.16	1.43	9.41	88.99	78	0.21
4	17.64	15.14	1.64	10.86	90.13	75	0.16

[a]0 represents using fresh slurry.

Supplementary Table 3: CO_2/N_2 gas mixture (z_1=0.2231) separation results by using ZIF-8/glycol slurry with a ZIF-8 mass fraction of 0.152 at nearly the same initial pressure of around 17.58 bar and different experimental temperatures.

T (K)	P_E (bar)	Φ	$P_{E\text{-}CO2}$ (bar)	y_1 (%)	x_1 (%)	β	S_c (mol L^{-1} bar^{-1})
274.15	15.10	50	1.24	8.20	92.92	147	0.27
283.15	14.98	46	1.33	8.89	95.38	212	0.22
293.15	15.00	45	1.52	10.10	89.54	76	0.18
303.15	15.65	44	1.98	12.67	81.06	29	0.12

Supplementary Table 4: CO_2/H_2 gas mixture (z_1=0.2386) separation results by using ZIF-8/glycol slurry with a ZIF-8 mass fraction of 0.152 at 293.15 K and different pressures.

P_0 (bar)	P_E (bar)	Φ	$P_{E\text{-}CO2}$ (bar)	y_1 (%)	x_1 (%)	β	S_c (mol L^{-1} bar^{-1})
7.33	6.18	18	0.72	11.62	98.99	745	0.15
9.28	8.12	23	1.03	12.71	98.14	362	0.16
12.43	10.52	31	1.02	9.68	95.47	197	0.22
17.25	14.36	43	1.24	8.66	95.46	133	0.26
27.62	23.82	70	2.59	10.87	92.34	99	0.18
36.85	32.38	94	3.94	12.17	88.54	56	0.14

Supplementary Table 5: CO_2/CH_4 gas mixture (z_1=0.2268) separation results by using ZIF-8/glycol slurry; ZIF-8 mass fraction in slurry and experimental temperature are respectively specified to 0.152 and 293.15 K except for the two labeled experimental runs.

P_0 (bar)	P_E (bar)	Φ	$P_{E\text{-}CO2}$ (bar)	y_1 (%)	x_1 (%)	β	S_c (mol L^{-1} bar^{-1})
7.32	6.19	18	0.79	12.83	82.14	31	0.13
7.89	6.48	20	0.71	11.01	79.83	32	0.17
10.22	8.57	26	1.01	11.82	83.19	37	0.14
13.66	11.06	34	1.21	10.96	74.19	23	0.17
18.33	15.30	48	1.95	12.74	70.51	17	0.13
25.26	21.28	67	2.84	13.35	69.94	15	0.12
27.52	23.29	73	3.17	13.60	70.90	15	0.11
10.19[a]	8.68	24	1.14	13.08	82.45	31	0.11
10.38[b]	8.76	24	1.00	11.43	82.56	37	0.14

[a] The experimental temperature and ZIF-8 mass fraction in the slurry were specified to 303.15 K and 0.152, respectively.

[b] The experimental temperature and ZIF-8 mass fraction in the slurry were specified to 303.15 K and 0.173, respectively.

Supplementary Table 6: CO_2/N_2 gas mixture (z_1=0.2231) separation results by using slurries formed by ZIF-8 with different liquids at 293.15 K.

Slurry	m_F	P_0 (bar)	P_E (bar)	Φ	$P_{E\text{-}CO2}$ (bar)	y_1 (%)	x_1 (%)	β	S_c (mol L^{-1} bar^{-1})
ZIF-8 + ethanol	0.275	18.01	15.57	66	2.24	14.41	64.62	11	0.14
ZIF-8 + cyclohexane	0.278	17.92	14.24	53	1.78	12.50	60.53	11	0.22
ZIF-8 + n-hexane	0.302	19.13	16.02	53	2.30	14.37	63.86	11	0.10
ZIF-8 + methylbenzene	0.257	22.88	20.64	89	3.46	16.76	75.98	16	0.08
ZIF-8 + tetrachloromethane	0.111	17.61	13.21	44	1.29	9.77	58.94	13	0.23
ZIF-8 + triethylene glycol	0.151	17.79	15.65	45	1.98	12.65	88.89	55	0.12

Supplementary Table 7: Experimental results in absorption separation of CO_2 containing gas mixtures using glycol or water at 293.15 K.

P_0 (bar)	P_E (bar)	Φ	$P_{E\text{-}CO2}$ (bar)	y_1 (%)	x_1 (%)	β	S_c (mol L^{-1} bar^{-1})
Feed gas: 22.3% CO_2 + 77.7% N_2; Solvent: glycol							
18.00	16.39	54	2.79	17.03	72.12	13	0.057
Feed gas: 22.7% CO_2 + 77.3% CH_4 ; Solvent: water							
12.59	11.85	39	2.32	19.61	50.05	4.1	0.037

Supplementary Table 8: CO_2/N_2 gas mixture (z_1=0.2286) separation results in solid ZIF-8 at 293.15 K with different initial gas-solid volume ratio (Φ') and initial pressure (P_0).

Φ'	P_0 (bar)	P_E (bar)	$P_{E\text{-}CO2}$ (bar)	y_1 (%)	x_1 (%)	β'
137	8.03	6.81	1.03	15.18	66.21	11
206	12.02	9.78	1.43	14.67	58.52	8.2
272	15.41	12.49	1.90	15.22	58.52	7.9
361	20.87	17.11	2.61	15.27	56.63	7.2
423	24.48	20.13	3.10	15.40	57.18	7.3
542	31.09	25.87	4.04	15.60	58.06	7.5

Supplementary Table 9: CO_2/H_2 gas mixture (z_1=0.2386) separation results in solid ZIF-8 at 293.15 K with different initial gas-solid volume ratio (Φ') and initial pressure (P_0).

Φ'	P_0 (bar)	P_E (bar)	$P_{E\text{-}CO2}$ (bar)	y_1 (%)	x_1 (%)	β'
123	7.28	6.28	0.90	14.39	86.43	37
190	11.24	9.62	1.38	14.36	81.55	26
297	18.09	14.81	2.15	14.50	75.08	18
352	21.24	17.64	2.55	14.45	76.77	19
435	26.01	21.97	3.21	14.60	77.81	20
525	31.72	26.53	3.91	14.72	76.64	19

Supplementary Table 10: CO_2/CH_4 gas mixture (z_1=0.2342) separation results in solid ZIF-8 at 293.15 K with different initial gas-solid volume ratio (Φ') and initial pressure (P_0).

Φ'	P_0 (bar)	P_E (bar)	$P_{E\text{-}CO2}$ (bar)	y_1 (%)	x_1 (%)	β'
157	9.16	6.23	1.09	17.42	36.58	2.7
230	13.44	9.48	1.64	17.29	38.80	3.0
323	18.41	13.52	2.38	17.60	39.49	3.0
419	23.48	17.77	3.13	17.62	40.98	3.2
520	28.72	22.20	4.00	18.02	41.11	3.2
588	32.08	25.40	4.60	18.12	42.33	3.3

Supplementary Table 11: CO_2/N_2 gas mixture (z_1=0.2065) separation results by using glycol-mIm liquid mixture where mass ratio between glycol and mIm in liquid and experimental temperature are specified to 3:2 and 303.15 K, respectively.

Φ	P_0 (bar)	P_E (bar)	$P_{E\text{-}CO2}$ (bar)	y_1 (mol%)	x_1 (mol%)	β	S_c (mol L^{-1} bar^{-1})
11	5.65	4.55	0.10	2.26	82.65	206	0.94
15	7.68	6.20	0.15	2.40	86.90	270	0.86
20	9.82	8.00	0.22	2.70	87.70	257	0.75
24	11.63	9.45	0.24	2.53	83.81	199	0.82

Supplementary Table 12: CO_2/N_2 gas mixture (z_1=0.2065) separation results by using ZIF-8/glycol-mIm slurry; ZIF-8 mass fraction in slurry, mass ratio between glycol and mIm, and experimental temperature are respectively specified to 0.15, 3:2 and 303.15 K expect for the labeled experimental run.

Φ	P_0 (bar)	P_E (bar)	$P_{E\text{-}CO2}$ (bar)	y_1 (mol%)	x_1 (mol%)	β	S_c (mol L^{-1} bar^{-1})
9	4.43	3.51	0.08	2.40	74.41	118	0.92
14	6.60	5.22	0.07	1.38	83.74	367	1.63
17	8.28	6.62	0.10	1.59	84.77	345	1.43
24	11.35	9.17	0.15	1.62	85.45	357	1.40
35	16.51	1.33	0.23	1.73	87.43	394	1.29
14[a]	6.70	5.40	0.12	2.25	81.23	188	0.92

[a] The temperature of this experimental run was specified to 313.15 K.

Supplementary Table 13: CO_2/N_2 gas mixture (z_1=0.2065) separation results by using ZIF-8/glycol-mIm slurry with three different ZIF-8 mass fractions (m_F) in slurry where mass ratio between glycol and mIm and experimental temperature are specified to 3:2 and 303.15 K, respectively.

m_F (%)	P_0 (bar)	P_E (bar)	$P_{E\text{-}CO2}$ (bar)	y_1 (mol%)	x_1 (mol%)	β	S_c (mol L^{-1} bar^{-1})
5	6.30	5.13	0.11	2.18	85.08	256	1.00
10	6.46	5.12	0.08	1.53	82.99	313	1.48
15	6.60	5.22	0.07	1.38	83.74	367	1.63

Supplementary Table 14: CO_2/CH_4 gas mixture (z_1=0.2193) separation results by using ZIF-8/glycol-mIm slurry where ZIF-8 mass fraction in slurry, temperature, mass ratio between glycol, and mIm are specified to 0.15, 3:2, and 303.15 K, respectively.

Φ	P_0 (bar)	P_E (bar)	$P_{E\text{-}CO2}$ (bar)	y_1 (mol%)	x_1 (mol%)	β	S_c (mol L^{-1} bar^{-1})
11	5.39	4.03	0.10	2.76	69.81	81	0.89
14	6.48	4.91	0.12	2.05	74.24	138	1.21
16	7.56	5.78	0.14	2.21	73.86	125	1.12
20	9.29	7.18	0.18	2.18	75.86	141	1.13
32	14.66	11.48	0.28	2.13	76.89	144	1.14
96	41.38	32.79	0.87	2.65	78.45	134	1.00
161	66.41	53.46	1.32	3.13	73.90	87	0.76

Supplementary Table 15: CO_2/H_2 gas mixture (z_1=0.2360) separation results by using ZIF-8/glycol-mIm slurry where ZIF-8 mass fraction in slurry, mass ratio between glycol and mIm, and temperature are specified to 0.15, 3:2, and 303.15 K, respectively.

Φ	P_0 (bar)	P_E (bar)	$P_{E\text{-}CO2}$ (bar)	y_1 (mol%)	x_1 (mol%)	β	S_c (mol L^{-1} bar^{-1})
13	6.40	4.95	0.10	2.09	86.04	288	1.23
23	11.33	8.91	0.17	1.88	88.08	386	1.37
44	21.51	16.99	0.32	1.88	92.23	618	1.33
65	31.60	25.38	0.47	1.85	94.25	871	1.35
85	41.33	33.41	0.68	2.02	95.16	951	1.21

Supplementary Table 16: Results of the breakthrough experiment for CO_2/N_2 mixture (z_1=0.2065) with inlet gas flow rate, V_{in}, of 23 (mL min^{-1}) in ZIF-8/glycol-mIm slurry.

Time (h)	N_2 in outlet gas (mol%)	outlet gas flow V_{out} (mL min^{-1})	[a]R_{N2} (%)	[b]R_{CO2} (%)
0	0	0.0	-	
0.008	100	6.0	32.88	100
0.10	100	10.0	54.79	100
0.28	99.56	14.0	76.37	98.70
1.38	98.24	16.0	86.13	94.07
1.90	97.68	16.5	88.31	91.94
2.11	97.36	17.0	90.69	90.55
2.26	97.07	18.0	95.74	88.90
2.48	96.73	18.2	96.46	87.47
2.74	96.38	18.5	97.70	85.90
2.92	95.89	19.0	99.83	83.56
3.14	95.65	19.5	102.20	82.14
3.31	95.28	19.7	102.85	80.42
3.55	94.94	20.0	104.04	78.69
3.80	94.66	20.2	104.77	77.29
4.06	94.13	20.5	105.73	74.66
4.33	93.86	20.7	106.46	73.24
4.60	93.19	20.9	106.72	70.03
4.86	92.79	21.0	106.77	68.12
5.15	92.32	21.1	106.73	65.88
5.41	92.00	21.3	107.37	64.12
6.06	91.63	21.4	107.44	62.29
6.70	90.65	21.7	107.78	57.28

[a] $R_{N2} = \frac{V_{in} \times y_{in-N2}}{V_{out} \times y_{out-N2}} \times 100$, R_{N2} is the transient recovery ratio of N_2 in the outlet gas phase, where y_{in-N2} and y_{out-N2} are the mole fraction of N_2 in the feed gas and outlet gas, respectively; R_{N2} with a value higher than 100% indicates that N_2 is desorbed from the system.

[b] $R_{CO2} = 1 - \frac{V_{in} \times y_{in-CO2}}{V_{out} \times y_{out-CO2}} \times 100$, R_{CO2} is the transient recovery ratio of CO_2 in the slurry, where y_{in-CO2} and $y_{out-CO2}$ are the mole fraction of CO_2 in the feed gas and outlet gas, respectively.

Supplementary Table 17: Results of breakthrough experiment for CO_2/CH_4 mixture (z_1=0.276) with inlet gas flow rate, V_{in}, of 24 (mL min^{-1}) in ZIF-8/glycol-mIm slurry.

Time (h)	CH_4 in outlet gas (mol%)	outlet gas flow V_{out} (mL min^{-1})	R_{CH4} (%)	R_{CO2} (%)
0.00	0.00	0.0	-	-
0.01	100.00	6.0	35.51	100
0.10	100.00	9.0	53.27	100
0.17	99.79	10.0	59.06	99.70
0.50	98.48	11.0	64.11	97.65
0.88	97.48	12.0	69.23	95.74
1.37	96.96	12.5	71.73	94.65
1.75	96.10	13.0	73.94	92.86
2.70	95.49	13.5	76.30	91.43
3.38	94.44	14.0	78.25	89.04
4.23	93.44	14.5	80.19	86.61
4.87	92.41	15.5	84.77	83.44
6.03	90.92	17.0	91.48	78.27
6.95	89.86	18.5	98.39	73.59
7.70	88.98	19.0	100.06	70.53
9.07	87.84	20.0	103.98	65.77
9.38	87.27	20.5	105.89	63.27

Supplementary Table 18: Results of the breakthrough experiment for CO_2/CH_4 mixture (z_1=0.276) with inlet gas flow rate, V_{in}, of 24 (mL min^{-1}) in pure water.

Time (h)	CH_4 in outlet gas (mol%)	outlet gas flow V_{out} (mL min^{-1})	R_{CH4} (%)	R_{CO2} (%)
0.00	0.00	0.0	-	-
0.001	100	6.0	34.09	100
0.005	99.79	10.0	56.70	99.68
0.13	99.15	13.0	73.23	98.33
0.23	97.50	13.5	74.79	94.90
0.32	94.81	14.5	78.11	88.64
0.43	89.33	16.0	81.21	74.23
0.56	81.48	18.5	85.64	48.28
0.73	76.33	20.5	88.91	26.75
1.02	74.48	21.5	90.98	17.17
1.22	74.33	22.0	92.91	14.74
1.43	74.18	22.5	94.83	12.30

Supplementary Table 19: Henry constant (*H*) of gas components in liquid glycol at 293.15 K.

Gas components	*H* (MPa g $mmol^{-1}$)
CO_2	2.15
CH_4	14.7
N_2	48.2
H_2	37.6

Supplementary references:

1. Pérez-Pellitero, J. *et al.* Adsorption of CO_2, CH_4, and N_2 on zeolitic imidazolate frameworks: experiments and simulations. *Chem. Eur. J.* **16**, 1560-1571 (2010).
2. Amrouche, H. *et al.* Experimental and computationalstudy of functionality impact on sodalite-zeolitic imidazolate frameworks for CO_2 separation. *J. Phys. Chem. C* **115,** 16425-16432 (2011).
